# Discharge Patterns of Potentially Harmful Elements (PHEs) from Coking Plants and Its Relationship with Soil PHE Contents in the Beijing–Tianjin–Hebei Region, China

**DOI:** 10.3390/toxics10050240

**Published:** 2022-05-10

**Authors:** Xiaoming Wan, Weibin Zeng, Gaoquan Gu, Lingqing Wang, Mei Lei

**Affiliations:** 1 Institute of Geographic Sciences and Natural Resources Research, Chinese Academy of Sciences, Beijing 100101, China; 2 University of Chinese Academy of Sciences, Beijing 100049, China

**Keywords:** accumulation, atmospheric deposition, emission, ecological risk, spatial heterogeneity

## Abstract

The Beijing–Tianjin–Hebei (BTH) region in China is a rapid development area with a dense population and high-pollution, high-energy-consumption industries. Despite the general idea that the coking industry contributes greatly to the total emission of potentially harmful elements (PHEs) in BTH, quantitative analysis on the PHE pollution caused by coking is rare. This study collected the pollutant discharge data of coking enterprises and assessed the risks of coking plants in BTH using the soil accumulation model and ecological risk index. The average contribution rate of coking emissions to the total emissions of PHEs in BTH was ~7.73%. Cross table analysis indicated that there was a close relationship between PHEs discharged by coking plants and PHEs in soil. The accumulation of PHEs in soil and their associated risks were calculated, indicating that nearly 70% of the coking plants posed a significant ecological risk. Mercury, arsenic, and cadmium were the main PHEs leading to ecological risks. Scenario analysis indicated that the percentage of coking plants with high ecological risk might rise from 8.50% to 20.00% as time progresses. Therefore, the control of PHEs discharged from coking plants in BTH should be strengthened. Furthermore, regionalized strategies should be applied to different areas due to the spatial heterogeneity of risk levels.

## 1. Introduction

China is the largest coke producer and consumer in the world. The annual coke production in China as of 2020 has reached 430 million tons, contributing to more than 60% of the world’s coke production [1]. The Beijing–Tianjin–Hebei (BTH) region, the Capital Economy Circle of China, is one of the main areas with concentrated coking plants. Coke production in BTH accounts for 12% of the whole country [1,2].

Studies have been conducted on the PHEs emission patterns from the coking industry. Copper (Cu), zinc (Zn), arsenic (As), lead (Pb), chromium (Cr), nickel (Ni), mercury (Hg), cadmium (Cd), and cobalt (Co) are the most abundant PHEs emitted from the coking industry and are commonly found in abundance in raw coal [3]. Although coking has been considered as a main contributor to PHE release in BTH [4,5,6], most studies concerning the coking industry focus on the emission patterns of organic contaminants [7,8,9], with less attention paid to PHE emissions. Until now, no official data on PHE emissions from coking plants in BTH have been available. The exact contribution of coking to the total PHEs released in BTH is still unknown, which is important for the government to establish regional policy.

Furthermore, during coking activities, PHEs are volatized, released, and then deposited on the surrounding soil [10,11,12,13]. Direct air pollutant emissions from iron and steel production plants account for 21% of the total suspended particulates emissions in the BTH region [14]. Atmospheric deposition is one of the main sources of PHEs in soil [15] and in crops [16]. Considering the intensive distribution of coking plants in BTH, coking has been regarded as one of the main sources of soil PHE pollution in this area. The apparent contribution of coking to soil pollution has also been identified in other areas of the world. Atmospheric emissions and dusting from the surface of ash dumps are essential sources for Cr, Mn, Ni, Cu, Zn, Cd, and Pb in soils around a coal-fired power station in Southern Russia [17]. The coke industry and steel metallurgy have been identified as sources of soil contamination by PHEs around the Zdzieszowice coking plant, Opole Province, Southern Poland [18].

There have been studies on the PHE pollution status in the soil around coking plants, which was found to be serious [19,20,21]. It has been found that PHEs in soil, especially As, Cd, and Hg, led to significant ecological risks, while Hg posed the most serious risk [22,23,24]. Furthermore, PHE emissions from coking plants may bring certain carcinogenic and non-carcinogenic risks to surrounding residents; and the health risks of As, Cr, and Ni are relatively high [25]. Moreover, soils with a high level of PHEs may result in the accumulation of PHEs in plants, which will reduce soil economic benefits and increase human health risks [15,26,27].

At a regional scale, coal combustion may lead to differences in the temporal and spatial distribution of PHEs, leading to the heterogeneous distribution of risks caused by coking plants [28,29]. Current risk assessments mostly focus on the soil pollution status inside a certain coking plant, with few considerations of the pollution from coking plants to the surrounding soil through atmospheric deposition of coking gas [30,31,32]. The lack of risk assessment at a regional scale may cause difficulties for the overall management of risks caused by coking plants in BTH. Therefore, quantifying the risks of PHE emissions from coking plants, and its spatial heterogeneity on a regional scale can provide valuable information to understand and effectively manage metal-induced risks [33,34].

This study aimed to disclose the discharge patterns of PHEs from coking plants and their relationship with soil PHEs contents in the BTH region. First, the data for PHEs discharged from coking plants in BTH were collected from the China Industrial Enterprise Pollution Emission Database [35], and POI data from Baidu map. Second, the data for PHE concentrations in soil were collected from published literature. Third, the relationship between the discharge data and soil concentration data was disclosed. Then, the ecological risks of PHEs accumulated from coking emissions were assessed. Scenario analysis was further conducted to predict the change in risk levels in the future given that PHEs could persist in soil for a very long time. The results could provide a quantified understanding of the degree of risks brought by coking plants in BTH. On this basis, possible suggestions on emission control were provided to protect the soil environment in the BTH region.

## 2. Materials and Methods

### 2.1. Study Area

**Figure 1:**
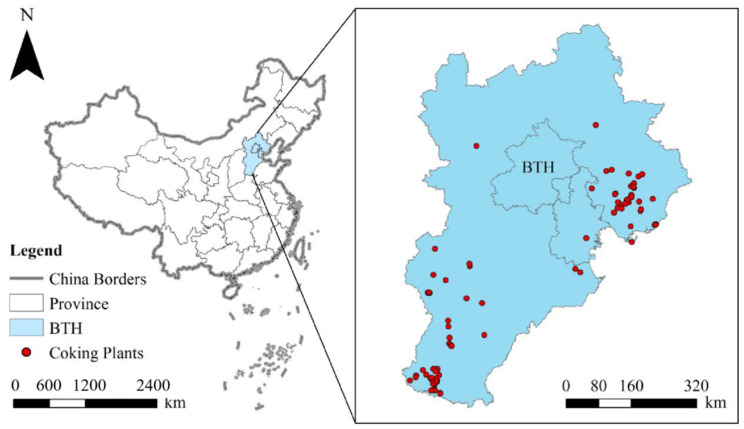
Location of coking plants in Beijing–Tianjin–Hebei Region, China.

The BTH region is in the northern part of the North China Plain close to the Northwest Pacific Ocean, and it is located between 113° E–119° E and 36° N–42° N (Figure 1). BTH are composed of 2 provincial-level municipalities (i.e., Beijing City and Tianjin City) and 11 prefecture-level cities, namely, Shijiazhuang (SJZ), Tangshan (TS), Qinhuangdao (QHD), Handan (HD), Hengshui (HS), Xingtai (XT), Baoding (BD), Zhangjiakou (ZJK), Chengde (CD), Cangzhou (CZ), and Langfang (LF). BTH cover an area of 218,000 km^2^ and have a population of approximately 110 million (National Bureau of Statistics in China, NBSC, 2019). The BTH region has a temperate monsoon climate with an average temperature of 3.9 °C~15.3 °C.

The terrain conditions of the BTH region are complex. The overall terrain is high in the northwest and low in the southeast. From northwest to southeast are situated Bashang Plateau, Yanshan Taihang Mountain and the southeast plain. Among them, the southeast plain can be divided into five types, namely, alluvial proluvial fan, flood plain, yellow flood plain, alluvial marine plain and marine plain. The ground elevation of Bashang Plateau, Yanshan Taihang Mountain, southeast plain, and the coastal areas is 1300–1700 m, 500–1000 m, less than 200 m, and less than 4 m, respectively.

The BTH region is located in the Haihe River Basin. The surface water system is well-developed and includes Yongding River, Daqing River, Ziya River, South Canal, Luanhe River and other rivers. BTH belong to the Yanliao stratigraphic division and North China Plain stratigraphic division of the Shanxi Hebei Shandong Henan stratigraphic region. The Cenozoic strata are well-developed, and most of the surface is Quaternary. The bedrock strata under the Quaternary system are basically complete, lacking a Neoproterozoic Sinian system, Paleozoic Silurian system and Devonian system. In the Cenozoic era, strong fault depressions and depressions were produced, and huge thick Cenozoic deposits were deposited.

The soil types of different geomorphic units are quite different. The main soil types in the Bashang plateau are calcium layer soil. The mountain area is mainly distributed with eluvial soil, semi eluvial soil, calcium layer soil and primary soil. The piedmont alluvial proluvial fan area is mainly semi eluvial soil. The flood plain and yellow flood plain are mainly distributed with semi hydrous soil. The marine plain is mainly developed with saline alkali soil.

### 2.2. Data Collection

#### 2.2.1. Data of Coking Enterprises and Their PHE Emissions

The data of the coking enterprises were mainly from two sources: one is the list of coking companies in production in BTH, and the other is the pollution emissions by coking companies in BTH. Coking enterprises in the current study included both coking enterprises and coking section of iron and steel industry.

The list of coking plants in production in BTH was obtained through POI data from Baidu map, which is the largest map search engine in China. The searching keyword including coking plant and steelworks. To ensure the accuracy of the data, we filtered the companies that meet the access conditions of the coking industry published by the Ministry of Industry and Information Technology of China from the companies list. A total of 80 coking plants were identified in BTH.

The data of pollution emission from each coking enterprise were obtained through the China Industrial Enterprise Pollution Emission Database [35]. This database was a unique and comprehensive firm-level database, established on the basis of Statistical Report Statistics of Industrial Enterprises above Designated Size by the National Bureau of Statistics in China [1]. This database provides information on the industrial output value, energy input and pollution emission of enterprises. The emission list of databases included the locations, gross industrial output value, coal consumption, emissions of particulate matter, and exhaust gas emissions of coking plants in production. The data were independently reported by polluting enterprises, counted by environmental protection departments, and finally monitored and irregularly inspected by local environmental protection departments at the county level to ensure data quality.

PHE emissions were calculated by combining coal consumption data with specific emission factors grouped by different boiler patterns and various air pollutant control device configurations [6,11,13], as shown in Table S1 (Supplementary Sile). The combustion emission factor data of As, Cd, Cr, Cu, Hg, Ni, Pb, and Zn of the coking plants were 1.64, 0.10, 3.60, 1.71, 0.17, 0.94, 2.22, and 7.55 μg·kg^−1^ [12].

#### 2.2.2. Coal Consumption by Enterprises in BTH

The coal consumption data of each enterprise were also obtained through the annual coal-burning data published by the China Industrial Enterprise Pollution Emission Database [35].

#### 2.2.3. Soil Environmental Quality Data in BTH

To understand the soil environmental status of the BTH region, data of the soil PHE concentrations in this region were collected from literature published from 2010 to 2020. Two databases were searched: the China Knowledge Resource Integrated Database (the main database for Chinese research) and Web of Science. The literature selection criteria were as follows: (1) the original pollutant concentrations and sampling locations could be obtained; (2) the sampling and analysis methods of pollutants should be of international or domestic standards to facilitate the comparison of results. The studied pollutants include arsenic (As), cadmium (Cd), zinc (Zn), chromium (Cr), mercury (Hg), copper (Cu), nickel (Ni), and lead (Pb). Data were obtained from about 60 sampling locations. Representative references are listed here [36,37,38,39,40,41].

### 2.3. Data Analysis

#### 2.3.1. PHE Emissions from Coal-Consuming Enterprises in BTH

PHEs emitted by coal consuming enterprises and coking industries in BTH were calculated based on the amount of coal, the content of PHEs in coal, and the release rates (Equations (1) and (2)). Proportion of PHEs discharged from coking plants in BTH was calculated by Equation (3).

(1) $$T_{i,\;j,\;\mathrm{BTH}}=A_{j,\;\mathrm{BTH}}\times C_{i,\;\;\mathrm{BTH}}\times r_{i}$$

(2) $$T_{i,\;j,\;\mathrm{Coke}}=A_{j,\;\mathrm{Cok}e}\times E_{i,\;C\mathrm{oke}}$$

(3) $$CR_{i,\;j}=T_{i,\;j,\;\mathrm{Coke}}/T_{i,\;j,\;\mathrm{BTH}}\times 100\%$$

where $T_{i,\;j,\;\mathrm{BTH}}$ is the total emission of PHE *i* in city *j* in BTH, mg; $A_{j,\;\mathrm{BTH}}$ is the coal consumption of city *j* in BTH, kg; $C_{i,\;\mathrm{BTH}}$ is the content of PHE *i* in coal in BTH, μg·g^−1^; $r_{i}$ is the release rate of PHE *i* from coal-consuming enterprises in BTH, %, shown in Table S1 (Supplementary Sile); $T_{i,\;j,\;\mathrm{Coke}}$ is the emission of PHE *i* from coking plants in city *j* in BTH, mg; $A_{j,\;\mathrm{Coke}}$ is the coal consumption of coking plants in city *j* in BTH, kg, obtained from China Industrial Enterprise Pollution Emission Database; $E_{i,\;C\mathrm{oke}}$ is the emission factor of PHE *i* during the coking process, being 1.64, 0.10, 3.60, 1.71, 0.17, 0.94, 2.22, and 7.55 μg·kg^−1^ for As, Cd, Cr, Cu, Hg, Ni, Pb, and Zn, respectively (USEPA (2008);$\;CR_{i,\;j}$ is the contribution rate of coking to all the coal-consuming enterprises, in terms of PHE *i* in city *j* in BTH [42].

#### 2.3.2. Relationship between PHEs Discharged from Coking Plants with the Soil PHE Concentrations in BTH

The Inverse Distance Weighted spatial interpolation tool of ArcGIS 10.2 was used to interpolate the data of soil environmental quality collected from the literature, obtaining the spatial distribution map of PHEs. Furthermore, using the value extraction to point extraction tool of ArcGIS, the interpolated PHE content corresponding to the location of those 80 coking enterprises is obtained. The correlation between the soil PHE concentrations in coking soil and the PHEs discharged from coking plants is analyzed using the cross-table analysis method of SPSS (SPSS 22.0).

#### 2.3.3. Soil Pollutant Accumulation from Coking Emissions

The soil pollutant accumulation model (Equations (4)–(7)) was used to calculate PHEs deposited on soil surrounding the coking plants [43].

(4) $$M_{i,\;\mathrm{emission}}=M_{\mathrm{coal}}\times F_{i}\times 10^{-3}$$

(5) $$W_{0}=M_{i,\;\mathrm{emission}}/V_{\mathrm{gas}}$$

(6) $$R=\;W_{0}\times V\times t/M$$

(7) $$W_{n}=RK1-K^{n}/\left(1-K\right)+C$$

where $W_{n}$ is the content of PHEs in the soil after n-year production of coking, mg·kg^−1^; $M_{i,\;\mathrm{emission}}$ is the total amount of PHEs *i* that were discharged by the coking plants, mg; $M_{\mathrm{coal}}$ is the coal consumption of the coking plant, kg·year^−1^; $F_{i}$ is the emission factor of PHE *i* during the coking process, μg·kg^−1^;$\;W_{0}$ is the maximum concentration of pollutants on the ground, mg·m^−3^; $V_{\mathrm{gas}}$ is the annual exhaust gas emitted from coking plants, m^3^; *R* is the annual input content of pollutants, mg·kg^−1^; *V* is the settlement rate, m·s^−1^; *t* is the annual settlement time, s; *M* is the mass of soil per square meter, kg/m^2^; *n* is the years of coking plants; *K* is the annual residual rate of PHEs in the soil, %; *C* is the background value of PHE content in BTH, mg·kg^−1^, obtained from a recently published national investigation [43]. The value of soil PHEs’ residue rate refers to the mentioned residue standard of 90%, the value of sedimentation rate is 0.001 m·s^−1^, and the value of soil mass is 169 kg [44].

#### 2.3.4. Calculation of Potential Ecological Risk

The potential ecological risk index method [27,45] was used to evaluate the potential ecological risks of soil PHEs obtained through sedimentation from coking waste gas in BTH (Equations (8) and (9)).

(8) $$E_{i}=Tr_{i}\cdot C_{i}/S_{i}$$

(9) $$RI=\sum_{i=1}^{n}E_{i}{\;}_{\;}$$

where $E_{i}$ is the potential ecological risk index of a single PHE *i*; $Tr_{i}$ is the toxicity coefficient of PHE *i*, and the coefficients of As, Cd, Cr, Cu, Hg, Ni, Pb, and Zn are 10, 30, 2, 5, 40, 5, 5, and 1, respectively [45,46,47]; $C_{i}$ is the concentration of a single PHE *i*, mg·kg^−1^; $S_{i}$ is a reference for a single PHE *i*, mg·kg^−1^, which was the background value of PHEs in BTH; *RI* is the comprehensive potential ecological risk index of PHEs. Risk classification standards refer to published studies [45,48] (Table S2, Supplementary File).

#### 2.3.5. Prediction of Ecological Risk Caused by Coking Plants

The accumulation of As, Cd, Cr, Cu, Hg, Ni, Pb, and Zn in the soil around the coking plants after 10, 20, 50, and 100 years of production was calculated [49], and compared with the thresholds of 30, 0.3, 200, 100, 2.4, 100, 120, and 250 specified by GB15618-2018 in China [50]. It was assumed that no soil remediation or discharge control measures were taken in this area, thereby remaining the same metals discharge and deposition ratio. The ecological risks posed by coking plants in BTH was calculated using Equations (8) and (9), based on the predicted PHE concentrations in the soil.

## 3. Results

### 3.1. Discharge Patterns of PHEs from Coking Plants

There are ~90 coking enterprises in the BTH region, mainly located in southeast regions, namely, Tangshan city and Handan city of Hebei Province (Figure 1). These two cities have about 30 coking enterprises in production, accounting for 67% of the coking enterprises in production in the BTH region.

The contribution rate of coking emissions of PHEs varied in different cities, and the average rate reached 7.73% (Table S3, Supplementary File). Among all the cities where coking plants were located, the order of contribution rate of the coking industry to the total PHE release was Xingtai (XT) > Tangshan (TS) > Handan (HD) > Baoding (BD) > Zhangjiakou (ZJK) > Chengde (CD) > Shijiazhuang (SJZ) > Tianjin (TJ) > Qinhuangdao (QHD). The contribution rates of PHEs in XT reached approximately 17%, and these rates were significantly higher than those of other cities. The contribution rates of coking plants to PHE emissions were higher in the southeast plain cities than in the northwest mountainous region.

**Figure 2:**
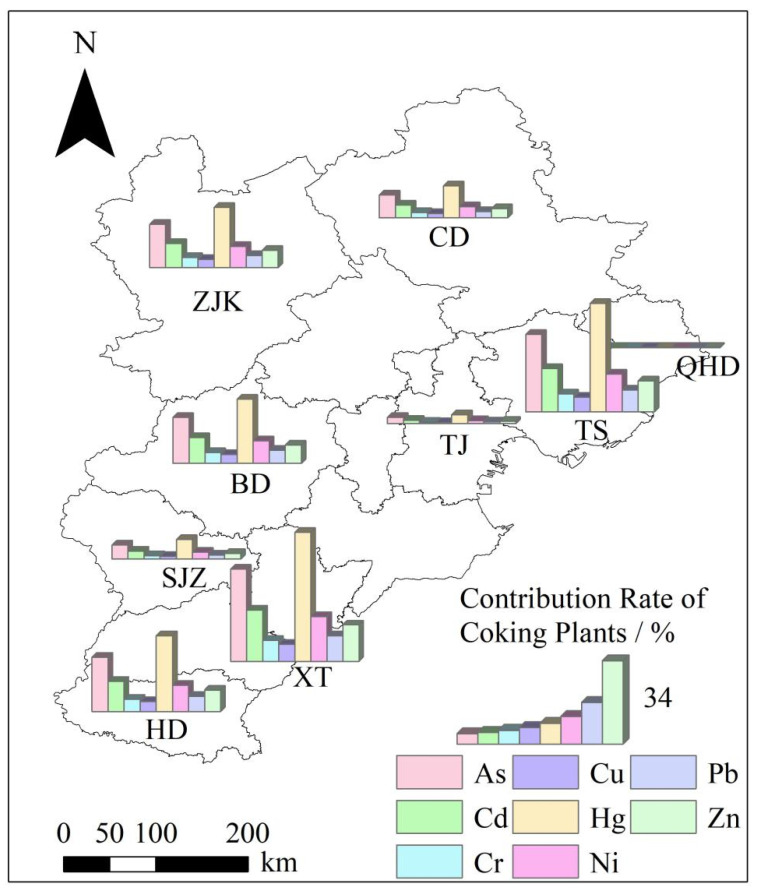
Contribution rate of coking plants to the PHE discharge in BTH. ZJK indicates Zhangjiakou, CD indicates Chengde, BD indicates Baoding, TJ indicates Tianjin, TS indicates Tangshan, QHD indicates Qinhuangdao, SJZ indicates Shijiazhuang, XT indicates Xingtai, and HD indicates Handan.

Regarding the contribution rate of a single PHE in a single city, the order of contribution rate of each PHE from high to low was Hg > As > Cd > Ni > Zn > Pb > Cr > Cu (Figure 2). The contribution rate of Hg in TS and XT exceeded 50%. In addition, the contribution rates varied among different elements and in different regions. Hg, As, and Cd showed a generally higher contribution rate from coking than other elements. The contribution rates of As and Cd in TS and XT nearly exceeded 20%.

### 3.2. Relationship between Soil PHE Concentration and PHE Discharge from Coking Plants in BTH

The overall soil environmental quality in the BTH region is relatively good, only As, Cr, Hg and Zn indicated a certain degree of over-screening ratio (Table 1).

**Figure 3:**
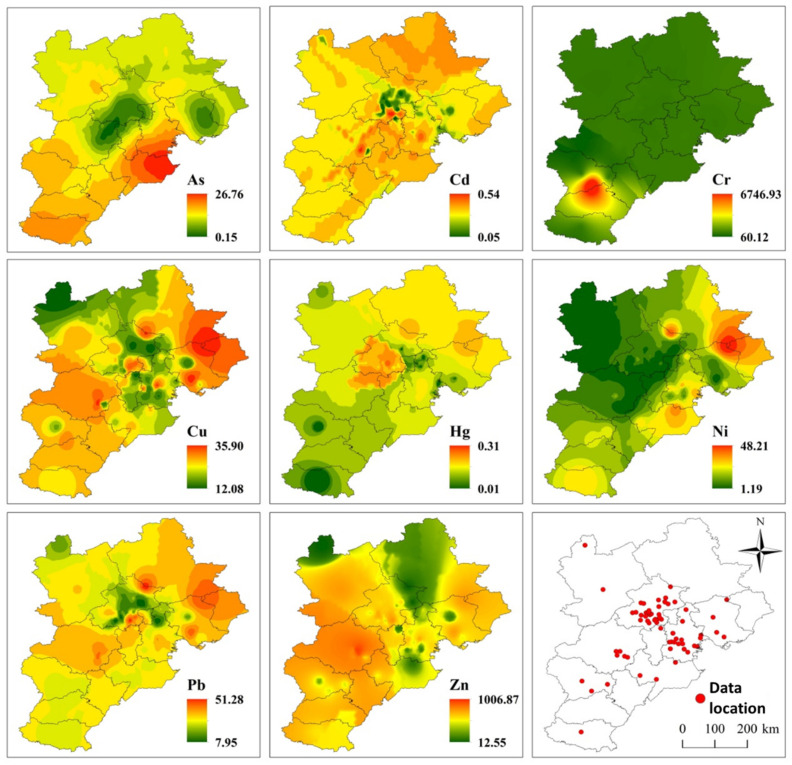
Distribution of PHE concentrations in BTH region (data were from published references).

The accumulation of PHEs in the southeast plain of BTH also presents certain local characteristics, in accordance with the distribution pattern of population and industry in this area. According to literature analysis, soil PHE pollution in the BTH region mainly occurs in the southeast plain, with no apparent soil PHE pollution in Yanshan Mountain in the north and Taihang Mountain in the west (Figure 3). Among them, the areas with high contents of As, Cd and Hg are mainly distributed along the coast of Bohai Bay. The contents of Cr, Zn and Cu are higher in Shijiazhuang, Xingtai and Handan in the south of Hebei Province, and the contents of Cu, Ni and Pb are higher in Tangshan and Qinhuangdao. Hg and Ni are also concentrated around Langfang city. Overall, the PHE pollution extent in the BTH region is low, showing a certain degree of local aggregation (Figure 3).

According to Table 2, the sig value of eight PHEs in soil quality corresponding to PHEs discharged by coking enterprises is greater than 0.05, and the phi value and V value are greater than 0.1, indicating that there is a close relationship between PHEs discharged by coking enterprises and the soil PHE concentration.

### 3.3. PHE Accumulation in Soil from Coking Emissions and Depositions, and Its Associated Ecological Risk

**Figure 4:**
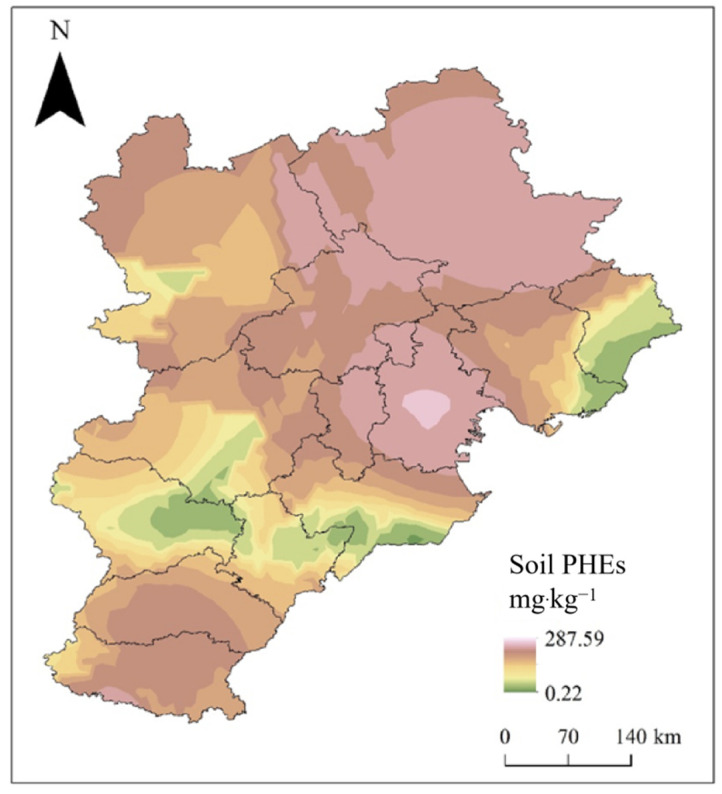
Sum of the eight PHEs after the discharge and deposition of PHEs from coking enterprises.

Excluding other pollution sources in the soil, the scenario simulation of PHEs in the soil around coking enterprises was carried out. Through the soil accumulation model, the distribution of soil PHEs in the BTH region after the discharge and deposition of PHEs from coking enterprises were calculated and depicted on a map (Figure 4), aiming to provide a general idea of PHE discharge on soil PHE accumulation.

The spatial distribution of PHEs in coking soil due to the deposition of PHEs discharged from coking waste gas at a regional scale of BTH indicated that the area with high PHEs in coking soil is located in the southeast and northeast of the BTH area (Figure 4), in accordance with the locations of coking plants in Figure 1.

**Figure 5:**
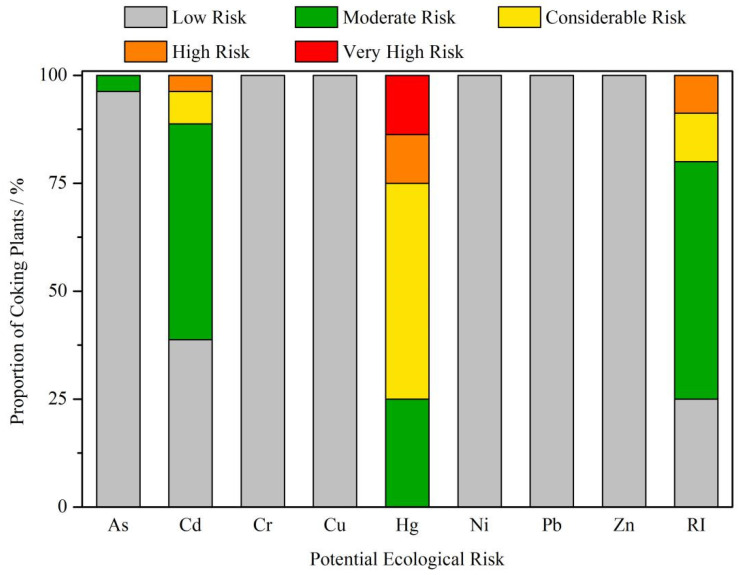
Proportion of coking plants causing ecological risk to the surrounding soil.

**Figure 6:**
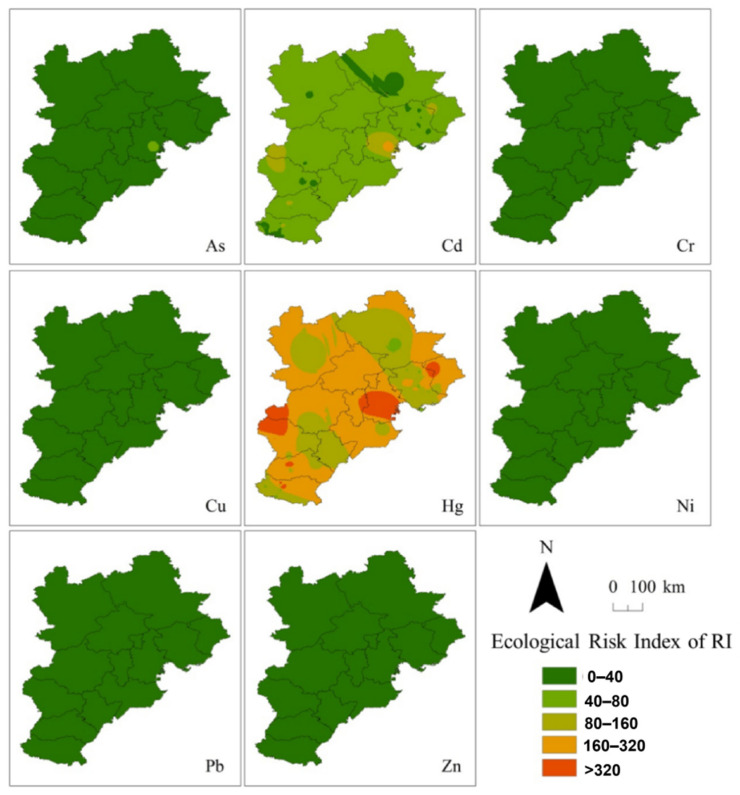
Distribution of RI index of coking plants causing potential ecological risk to the surrounding soil for each single PHE.

The ecological risks of soil PHEs resulting from coking were further assessed to provide suggestions for management in BTH (Figure 5 and Figure 6). Table 3 shows the potential ecological risk index E_i_ of a single PHE and comprehensive ecological risk index RI of multiple PHEs after the production period. The order of coefficient of variation of E_i_ (CV) was Hg > Cd > As > Pb > Ni > Cu > Zn > Cr (Table 3). Strong significant spatial variation of ecological risk was observed, especially for Hg, Cd, and As (Figure 6).

Figure 5 shows the comparative analysis results of potential ecological risk index E_i_ of PHEs and comprehensive ecological risk index RI of multiple PHEs. Hg, Cd, and As showed apparent ecological risk, while other PHEs had slight risk. In terms of the comprehensive potential risk, coking plants with high risk level accounted for 8.75% of the total coking plants, while coking plants with low risk level only accounted for around 25.00%. For As, 3.75% of the coking plants reached the moderate risk level, while the risk level of the remaining plants was low. For Cd, only 38.75% of the coking plants had low risk level, while 3.75% of coking plants reached high risk. For Hg, all enterprises reached a certain level of risk: 50% of the coking plants reached the moderate risk level, 11.25% of the coking plants reached the high-risk level, and 13.75% of the coking plants reached the very high-risk level.

**Figure 7:**
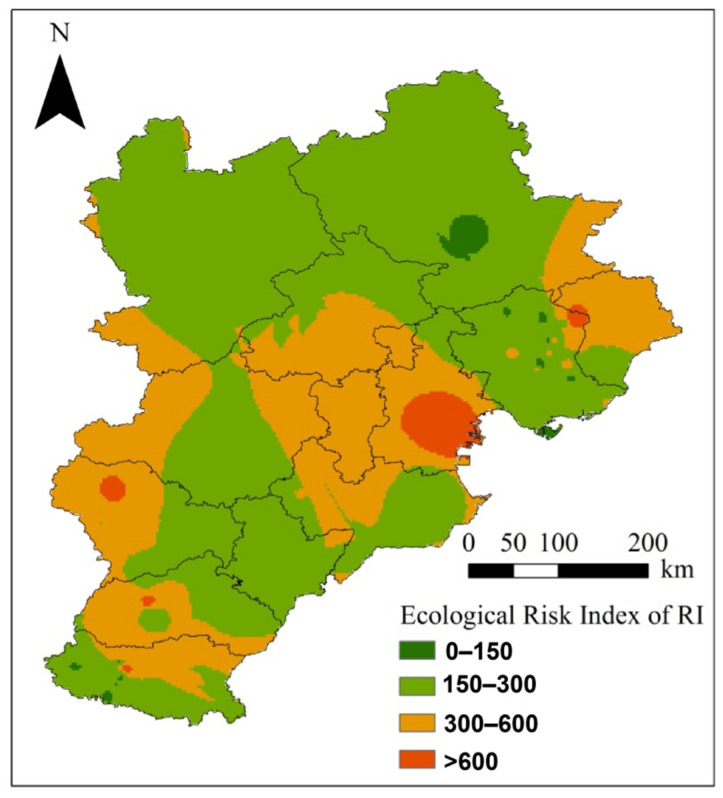
Distribution of RI index of coking plants causing potential ecological risk to the surrounding soil for all the PHEs.

In terms of the spatial distribution of comprehensive ecological risks (Figure 6 and Figure 7), some cities in the northern areas were at a lower risk level, while the remaining cities affected by PHE pollution emissions from coking plants were at a moderate risk or above. The results suggested higher risks from Hg, Cd, and As than other elements. One of the reasons is that the three elements had a higher emission than other metals (Figure 2). The higher toxicity of the three elements could also contribute to their high risks.

### 3.4. Prediction of the Potential Ecological Risk in the Future by Scenario Analysis

**Figure 8:**
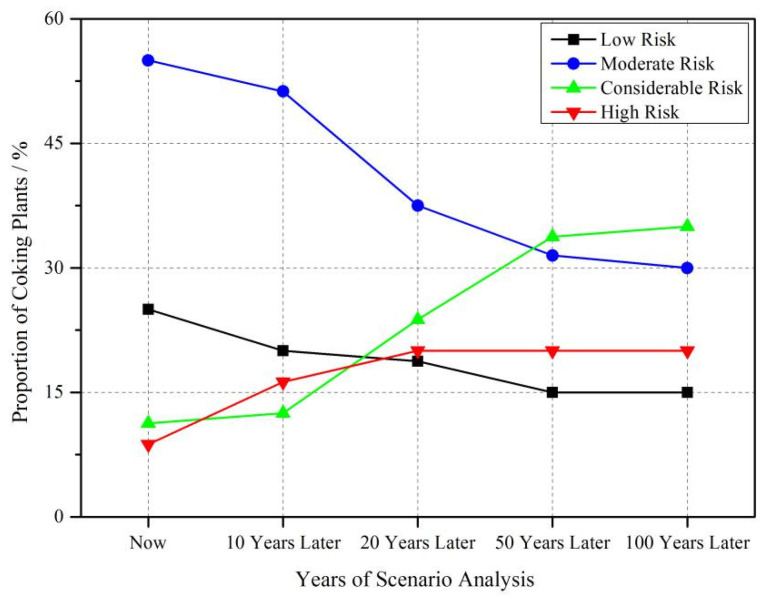
Scenario analysis of potential ecological risk of each coking plant in BTH.

Results indicated that after 10, 20, 50, and 100 years of production, some PHEs will exceed the threshold value after a certain period (Figure 8). As, Cd, and Zn will cause the surrounding soil to exceed the threshold in most coking plants. Hg, Ni, and Pb will also cause the surrounding soil to exceed the threshold in a small number of coking plants. The potential ecological risk index RI of the corresponding year and the proportion of coking plants of each risk level in different years were calculated on the basis of the simulated soil PHE content after years of coking production.

As the production time progressed, the proportion of coking plants with low and moderate risks gradually decreased, and the proportion of coking plants with considerable and high risks gradually increased. This finding indicates that the degree of risk continued to increase as production time progressed, and the trend of changes will be more serious. Notably, the role of considerable and moderate risks kept increasing as production time progressed. This result indicates that the coking plants presenting high risks should be under strict control.

## 4. Discussion

### 4.1. Coking Was an Important Source for the PHE Discharge in BTH

This report is the first to quantify the discharge pattern of PHEs from coking plants, and their contribution to the total PHE discharge in different districts of the BTH region. Despite the general idea that coking could be an important source for PHE discharge in BTH, there has been no quantitative analysis on the contribution rate. Through the current study, it was disclosed that coking plants also played an important role in discharging PHEs into the environment, other than discharging organic pollutants [51].

The contribution rate showed an apparent spatial heterogeneity (Figure 2), which might be related to the geomorphological characteristics of the BTH regions. The BTH region is located at the junction of coastal and inland areas in the middle latitude. The terrain is high in the northwest and low in the southeast. It transits from the northwest “Yanshan Taihang Mountain System” structure to the southeast plain. A considerably higher number of coking plants were located in the southeast region than other regions (Figure 1). Another report has also identified that pollution-intensive industries mainly concentrated in the southeast regions of BTH [52].

Another reason may come from the different categories of the main coal-consuming enterprises in different cities. The key coal consuming enterprises in BTH region included thermal power and coking and steel industries. The thermal power industries in TJ, SJZ, and TS consumed a considerably higher amount of coal than that in other cities. By contrast, the coking and steel industry in TS and HD consumed the highest amount of coal among all the cities in BTH. Due to the more intensive distribution of coking plants in XT, HD, and TS, the contribution rates of coking plants to the total PHE discharge in this area were higher.

In accordance with the current study, an investigation on the concentration of PHEs in PM_2.5_ in BTH also found that high emissions of PHEs are mainly distributed in the central and southern BTH region, especially for Beijing, Tianjin, SJZ, TS, HD and BD cities [53]. This may be attributed to the significant coal consumption of industrial boilers in the iron and steel industries, which are intensive and flourishing in XT, HD, and TS.

In terms of the difference among elements, Hg, As and Cd indicated a higher discharge ratio. Hg and As are comparatively volatile, which led to a higher contribution rate [54,55]. Results were in accordance with an earlier study, which also suggested that the east and south-central regions of BTH may have higher emissions of Hg, As, and Cd than other regions [6]. An earlier study also confirmed a considerable amount of atmospheric As, Pb, and Cd in BTH [56], indicating that the highest concentration of atmospheric As at the BD site was of 59 ng m^−3^; this value was 10 times the WHO standard (6.6 ng m^−3^). The value was lower in other cities of BTH, but it was still 2–3 times higher than the WHO limits. It has been suggested that other than the interprovincial pathways, the contribution of local coal burning and the iron industry to the pollution of aerosol trace elements in the region should be given more attention. Consideration should especially be given to the topographic features of the BTH region; anthropogenic emissions in BTH region were trapped in the boundary layer by the surrounding mountain ranges [57], which led to a higher deposition rate of atmospheric PHEs.

### 4.2. Coking Contributed to the Accumuation of PHEs in Soil

The meta-analysis on the PHE concentration in soil indicated that the overall soil environmental quality in BTH is good, with only As, Cd and Hg showing an accumulation along the coast of Bohai Bay. This was in accordance with the literature, suggesting the Tianjin coastal area was one of the high-risk regions in China [52]. The coastal zone is usually a diverse and complex system subjected to diversified anthropogenic activities, which is pronounced in the enrichment of aquatic soils with a number of metals from different types of sources that likely can be of both local and whole basin scale [29].

Comparing Figure 1, Figure 2 and Figure 3, it can be found that areas with a high content of soil PHEs, such as the coast of Bohai Bay and the south of Hebei Plain, also have more coking enterprises. This implies that the PHEs discharged by coking enterprises in Beijing, Tianjin and Hebei have a certain impact on the soil quality of Beijing, Tianjin and Hebei. The cross-table analysis further confirmed the relationship between coking emission and soil PHE concentrations.

The potential relationship between soil PHE concentration and PHE discharge from coking plants in BTH has been indirectly implied in the literature. Although also dependent on other sources such as runoff or soil additives, the PHE concentrations of topsoil was contributed significantly by atmospheric deposition [15]. In terms of the sources of PHEs in atmospheric deposition, coal burning was one of the most essential sources in BTH region [58,59].

The contribution of coking to soil PHE pollution is not only restricted to the BTH area but also exists in other areas of China, and even the world. Similar to the current study, in several steel plant areas, it has been found that PHE pollution in soil was the most serious in the coke processing plant, mostly from coke processing [60,61]. These reports have found that Hg and Pb were two of the main pollutants contributing to soil PHE pollution in this area, posing both high concentration and high bioavailability. One reference indicated that in the vicinity of coking plants, the water-soluble fraction of PHEs such as As, Cd and Sb reached as high as 50%, posing significant ecological risk and human health risks [24]. Due to the atmospheric emissions and dusting from the surface of ash dumps, Ni, Cd, and Pb were accumulated in soils around a coal-fired power station in Southern Russia and caused carcinogenic risk to human health [17]. Around a coking plant in Southern Poland, higher amounts of PHEs were also detected in soil [18].

### 4.3. Ecological Risk Analysis and Its Environmental Implications

The risk assessment results indicate that coking plants with moderate to high risk accounted for ~75% of the total coking plants. Scenario analysis indicates that if no measures are taken, the degree of risk might continue to increase, implying that the potential pollution will be more serious. Additionally, the high risks mainly come from Hg, Cd, and As, due to their higher emission (Figure 2) and higher toxicity [62] than other elements. Another study assessed the potential ecological risks of PHEs in topsoil of Shijiazhuang city in the BTH area, which showed that high concentrations of Cr, Cd and Ni distributed in the northeast of the urban area was related to the existence of steel and coking plants in that area [63]. They also spotted widespread contamination of Cd and Hg in the Shijiazhuang urban area, posing significant ecological risk. This similar trend between the current study and the study of Zou et al. [63] confirmed the non-negligible contribution of coking to the accumulation of PHE in soil. Furthermore, considering the potential interaction between PHE and organic pollutants co-released by coking plants [64], the PHE contamination surrounding the coking industry in the BTH area should be paid more attention.

The North China Plain is the most populous plain in China and forms the core of the BTH economic circle. With urbanization, anthropogenic PHEs have increasingly dispersed and accumulated in urban topsoil. Despite the generally known contribution of the coking industry to PHE emissions, attention has been mainly paid to polycyclic aromatic hydrocarbons [31,65]. Through the current study, the contribution of coking plants to PHE emissions in BTH was quantified, which reminded the related governor or scientist that As and Cd from coking plants should be paid more attention, especially considering their wide distribution and persistence in soil [66]. Furthermore, diversified control strategies should be applied to areas with different risk levels considering the apparent heterogeneity of risk levels in different cities of BTH. The emission and pollution control of coking plants in the southeast and northeast regions of BTH needs to be strengthened.

### 4.4. Uncertainty Analysis

There are limitations of the current study. This study only considered the PHE emissions of coal-consuming enterprises above a designated size in BTH. As a result, the total PHE emissions may be lower than the actual emissions. In addition, this study selected the coking emission factors based on EPA standards, given that no emission factors for separate elements are available in China. The emission factors may have differences due to the different production processes between the two countries [3]. The accumulation of PHEs and their potential ecological risks were determined by multiple factors [16].

When evaluating the correlation between soil PHE content and PHE discharge from coking plants, we only obtained 60 detailed sampling locations from the literature, therefore the spatial interpolation results may not be very accurate. Furthermore, the interpolation results were extracted from 80 coking enterprises to obtain the soil PHE concentration of coking plants. This data calculation could lead to inaccuracy.

The risk assessment of PHEs in soil was conducted under the assumption that PHEs in coking exhaust gas were all deposited in the location soil of each coking plant, without considering the transport of PHEs through dry or wet precipitation, and the associated spatial heterogeneity of PHE distribution. This might lead to the inaccurate evaluation of the spatial distribution of ecological risks. Further analysis on the temporal or spatial heterogeneity of the long-range transport of PHEs discharged from coking plants, especially [67], should be able to further improve our research. Evaluation of pollutant emissions and their interpretations from the aspects of spatial distribution is important for the proposal of pollution control measures [6].

During the prediction of risks caused by PHE emissions from coking plants in the future, the emission rate was assumed to be kept the same, and the soil also kept the status quo without remediation or a development plan. However, the truth of this case is yet to be seen. Therefore, the prediction results in the current study might not be accurate enough.

Despite these uncertainties, this report firstly quantified the contribution of the coking industry to the total PHE discharge in BTH through the extensive collection of pollutant discharge data and data calculation via the soil accumulation model and ecological risk index. Using the cross-table analysis, a close relationship was identified between the PHEs emitted by coking and the soil PHE concentration in the BTH region. Furthermore, at a regional scale, the potential ecological risk coefficient of PHEs discharged by coking was calculated to provide some feasible suggestions for the zoning of potential ecological risks of coking enterprises and the management of waste gas discharged by coking enterprises. This research presents a generalized pattern for the PHEs discharged from coking plants in BTH and its associated risk assessment and provides information for the regional pollution control. The results could, on the one hand, provide information for the diffusion pattern of coking pollutants, and, on the other hand, aid the government in controlling the potential risks caused by coking plants in BTH.

## 5. Conclusions

The contribution rates of coking plants to the total discharge pattern was firstly disclosed as being ~7.73%. Furthermore, this discharge of PHEs from the coking industry was found to be closely related to the accumulation of PHEs in soil. The discharge of Hg, As, and Cd from coking in BTH were relatively higher than those of other metals, which resulted in higher ecological risks of Hg, As, and Cd in this area. Other than the difference among metals, spatial heterogeneity should also be noticed. The eastern and central-southern parts of BTH showed high coking contribution rates. Most coking plants in eastern and central southern parts of BTH were at a moderate risk level. Scenario analysis also showed that the level of ecological risks would increase rapidly with an extending production time, especially for As, Cr, Ni, and Pb. Despite some uncertainties regarding the results, the regional-scale assessment of risks caused by coking plants could produce some suggestions for the government to better control the potential pollution due to coke production in BTH.

## Figures and Tables

**Table 1:** Statistics of PHE content in the BTH under existing research (mg·kg^−1^).

PHE	Max (mg·kg^−1^)	Min (mg·kg^−1^)	Screening Value (mg·kg^−1^) *	Over-Screening-Value Ratio (%)
As	27.6	4.70	20	4.55
Cd	1.45	0.06	20	0.00
Cr	6929	14.6	200	1.52
Cu	85.0	12.5	2000	0.00
Hg	1.62	0.01	8	2.38
Ni	69.0	12.0	150	0.00
Pb	117	2.15	400	0.00
Zn	1670	30.0	250	6.25

* Screening value refers to Soil environmental quality—Risk control standard for soil contamination of develop land (GB 36600-2018).

**Table 2:** Crosstabs analysis between PHEs discharged by coking and air quality.

PHE in Soil	PHE Released by Coking Plant		
	Sig.	Phi Value	V Value
As	0.252	8.367	1.000
Cd	0.364	4.243	1.000
Cr	0.252	8.367	1.000
Cu	0.367	4.123	1.000
Hg	0.367	4.123	1.000
Ni	0.252	8.367	1.000
Pb	0.283	7.141	1.000
Zn	0.367	4.123	1.000

**Table 3:** Descriptive Statistics of Cumulative Ei and RI of Coking Plants in BTH (N = 80).

Index of Risk	Maximum	Minimum	Mean	Standard	CV *
As-Ei	52.29	10.03	15.30	7.88	51.48
Cd-Ei	209.57	30.14	52.51	33.44	63.69
Cr-Ei	2.50	2.00	2.06	0.09	4.52
Cu-Ei	11.03	5.00	5.76	1.12	19.51
Hg-Ei	1199.75	40.88	185.36	216.00	116.53
Ni-Ei	12.69	5.01	5.96	1.43	24.00
Pb-Ei	17.21	5.01	6.53	2.27	34.82
Zn-Ei	2.52	1.00	1.19	0.28	23.82
RI	1507.55	99.07	274.68	262.53	95.58

* CV is the abbreviation for coefficient of variation.

## Data Availability

Data available on request from the authors.

## Supplementary Materials

The following supporting information can be downloaded at: https://www.mdpi.com/article/10.3390/toxics10050240/s1, Table S1: Averaged Concentrations (μg·g^−1^) and Emission Rates (%) of PHEs from Coals in the BTH, Table S2: Classification of Potential Ecological Risk Index, Table S3: Contribution Rate of Coking Plants to the Total PHEs Emissions in BTH.
