# Supplementary material for: Discharge Patterns of Potentially Harmful Elements (PHEs) from Coking Plants and Its Relationship with Soil PHE Contents in the Beijing–Tianjin–Hebei Region, China
Source: Toxics. 2022 May 10;10(5):240. doi: 10.3390/toxics10050240 (PMC9144211; doi:10.3390/toxics10050240)
Supplement: Supplementary file 1 [file toxics-10-00240-s001.zip › toxics-1705088-supplementary.pdf]

toxics

Supplementary Materials: Discharge Patterns of Potentially Harmful Elements (PHEs) from Coking Plants and Its Relationship with Soil PHE Contents in the Beijing–Tianjin–Hebei Region, China

Xiaoming Wan, Weibin Zeng, Gaoquan Gu, Lingqing Wang and Mei Lei

Table S1. Averaged Concentrations (μg·g^{-1}) and Emission Rates (%) of PHEs from Coals in the BTH [11,13].

Province	Hg	As	Pb	Cd	Cr	Ni	Cu	Zn
Beijing	0.17	4.02	26.74	0.66	23.39	15.27	27.82	63.24
Tianjin	0.17	3.98	26.52	0.69	22.39	15.14	27.84	63.76
Hebei	0.19	5.17	27.45	0.25	20.71	10.50	22.95	49.06
Emission rates	91.63	70.33	61.30	62.23	54.13	36.45	50.33	53.28

Table S2. Classification of Potential Ecological Risk Index.

Risk Level	Ei	Risk Level	RI
Low Risk (I)	0–40	Low Risk (I)	0–150
Moderate Risk (II)	40–80	Moderate Risk (II)	150–300
Considerable Risk (III)	80–160	Considerable Risk (III)	300–600
High Risk (IV)	160–320	High Risk (IV)	＞600
Very High Risk (V)	＞320		

Table S3. Contribution Rate of Coking Plants to the Total PHEs Emissions in BTH.

City	TJ	TS	HD	BD	CD
Percentage / %	1.15	14.15	9.93	8.36	4.19
City	SJZ	QHD	XT	ZJK	BTH
Percentage / %	2.57	0.05	16.82	7.87	7.73
